# Terrestrial Nocturnal Roosting Behavior of Black‐necked Cranes (*Grus nigricollis*) on the Yunnan‐Guizhou Plateau: Active Choice or Forced Environmental Adaptation

**DOI:** 10.1002/ece3.71485

**Published:** 2025-06-13

**Authors:** Xinlei Hou, Guangyi Lu, Shuxia Zhang, Liping Feng, Guopeng Ren, Heqi Wu

**Affiliations:** ^1^ College of Agriculture and Biological Science Dali University Dali China; ^2^ Kunming Institute of Zoology Chinese Academy of Sciences Kunming China; ^3^ School of Life Sciences and Engineering Henan University of Urban Construction Pingdingshan China; ^4^ Institute of Eastern‐Himalaya Biodiversity Research Dali University Dali China

**Keywords:** black‐necked crane, GPS‐GSM tracking device, nocturnal roosting sites, terragenization

## Abstract

Nocturnal roosting sites are integral to bird habitats, with their use and selection by birds serving as indicators of behavioral adaptations to environmental pressures. Black‐necked Cranes ( *Grus nigricollis* ), which typically roost in shallow water, have exhibited an unexpected “terrestrialization” of nocturnal roosting sites within their eastern wintering population of southwest China. Despite this phenomenon being documented since the late 20th century, research on terrestrial nocturnal roosting behavior remains limited, hindered by technological challenges. To address this knowledge gap, we combined GPS‐GSM tracking data from 14 individuals monitored between 2015 and 2022 in northeastern Yunnan and western Guizhou with remote sensing imagery to systematically analyze their nocturnal roosting patterns. Our results indicated that area of water body, the location of foraging grounds, and individual behaviors influenced the proportion of terrestrial nocturnal roosting in Black‐necked Cranes. On land, Black‐necked Cranes preferred to roost on highlands (headwaters, uphill terraces, mountain tops, and local ridges) and avoided valleys (canyons, shallow valleys, and U‐shaped valleys). Notably, nocturnal terrestrial roosting sites were associated with increased nocturnal mobility compared to shallow water (11.6% vs. 0.8%). These findings suggest that terrestrial roosting behavior may reflect adaptive trade‐offs under habitat pressure. We recommend that regional conservation strategies should prioritize the following: (1) Protect existing large wetlands, (2) Connect and restore fragmented small wetlands, (3) Strengthen nighttime monitoring of the Black‐necked Crane, and (4) Strictly manage free‐ranging dogs to minimize anthropogenic disturbance on terrestrially roosting cranes.

## Graphical abstract

The choice of roosting sites is crucial for organisms. Our results indicate that the Black‐necked Crane's terrestrial roosting behavior may reflect adaptive trade‐offs under habitat pressure.

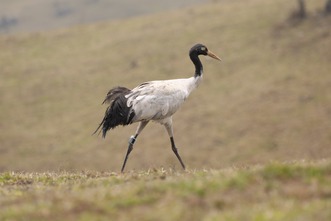

## 1 Introduction

Nocturnal roosting sites are a critical component of avian habitats. Suitable nocturnal roosting environments significantly influence the distribution, population density, and stability of bird populations (Masse 2014; Jirinec et al. 2016). Birds demonstrate clear preferences and selectivity in choosing nocturnal roosting sites, reflecting their behavioral adaptations to environmental pressures (Chamberlain et al. 2000; Boyce et al. 2002; He et al. 2011). Understanding nocturnal roosting sites provides valuable insights for bird conservation and management, as it is essential for analyzing avian ecological behavior, habitat selection, and population dynamics (Engel and Young 1992). Thus, the quality and characteristics of nocturnal roosting sites are fundamental to the study of avian nocturnal roosting behavior.

Among 15 extant crane species, the Gray Crowned Crane ( *Balearica regulorum* ) and the Black Crowned Crane ( *Balearica pavonina* ) uniquely roost nocturnally in trees (Johnsgard 1983a; Meine and Archibald 1996). In contrast, the majority of crane species typically roost in shallow aquatic environments, such as marshes, mudflats, shallow banks, and broad rivers with short vegetation, which are relatively free from disturbances (Johnsgard 1983b; del Hoyo 1996). The Black‐necked Crane ( *Grus nigricollis* ), discovered by humans last among crane species (Li and Li 2005), is unique due to its high‐altitude habitat and remains one of the least studied species (F. Li 2014). The Black‐necked Crane holds significant conservation status as a national Class I protected species in China, while globally, it is listed as Near Threatened (NT) by the International Union for Conservation of Nature (IUCN 2020). According to migration patterns and wintering habitats, the Black‐necked Crane is classified into three distinct wintering populations: the eastern, central, and western populations (Wu et al. 1993). The eastern population mainly breeds in the Ruoergai Wetland in southern Gansu and northwestern Sichuan in China. They primarily overwinter in three national nature reserves and their adjacent areas: Dashanbao and Huize in northeastern Yunnan, and Caohai in western Guizhou (L. Li 1997).

Field surveys indicate that overwintering Black‐necked Cranes typically select shallow water areas as nocturnal roosting sites (Li and Li 2005), including vegetated swamps (He et al. 2011), slow‐moving river branches (Tsamchu et al. 2014), and shallow regions of reservoirs and lakes (Chen 1997; Peng et al. 2013). However, a notable behavioral exception emerged when Qiu et al. (1995) first observed the eastern population's increasing use of terrestrial roosts in northeastern Yunnan, with cranes selecting leeward hillsides and valleys in locations that varied with wind direction. Subsequent studies by Wu et al. (2013) and Lu et al. (2017) confirmed this terrestrial roosting tendency. The terrestrial nocturnal roosting behavior of the eastern population of Black‐necked Cranes during the wintering period is uncommon among cranes. Wu et al. (2013) and Lu et al. (2017) speculate that this phenomenon is caused by the disappearance of shallow water areas and a large amount of human disturbance, but whether there are other reasons why Black‐necked Cranes choose to roost on land at night remains unclear.

The study of terrestrial nocturnal roosting sites of Black‐necked Cranes has significantly contributed to understanding their roosting ecology. However, the effectiveness of conventional field observations is compromised by three critical constraints: site accessibility barriers, nocturnal visibility restrictions, and meteorological interferences, all of which collectively impede systematic evaluation of roosting habitat preference mechanisms. Recent advances in 3S technologies (GIS, GPS, and RS) allow researchers to overcome these constraints through integrated satellite tracking and high‐resolution remote sensing. Satellite trackers now provide near‐real‐time animal location data (Lashley et al. 2018), enabling identification of nocturnal roosting sites via continuous nighttime positioning analysis. Integration with remote sensing imagery facilitates extraction of roosting site characteristics. Analysis of nocturnal movement distances and trajectories from continuous location points reveals activity levels reflecting nocturnal roosting site safety and disturbance resistance (Ordiz et al. 2017). To evaluate potential differences in disturbance resistance between shallow water and terrestrial nocturnal roosting sites, we calculated nocturnal movement distances across roosting types. This provides critical data for assessing nocturnal roosting site quality, making terrestrial nocturnal behavior investigation in the eastern wintering population a core focus of this study.

This research aims to identify the factors influencing the Black‐necked Cranes' choice of terrestrial nocturnal roosting sites, as well as to characterize these sites. Specifically, this study addresses the following questions: (1) What are the selection ratios of terrestrial nocturnal roosting sites in various wintering sites (e.g., Dashanbao, Huize, Caohai, etc.) for Black‐necked Cranes, and what factors influence these selection ratios? (2) What are the defining characteristics of nocturnal roosting sites in areas with a high prevalence of terrestrial nocturnal roosting? (3) Do terrestrial nocturnal roosting sites exhibit higher nocturnal mobility compared to shallow water nocturnal roosting sites?

## 2 Materials and Methods

### 2.1 Study Area

**FIGURE 1:**
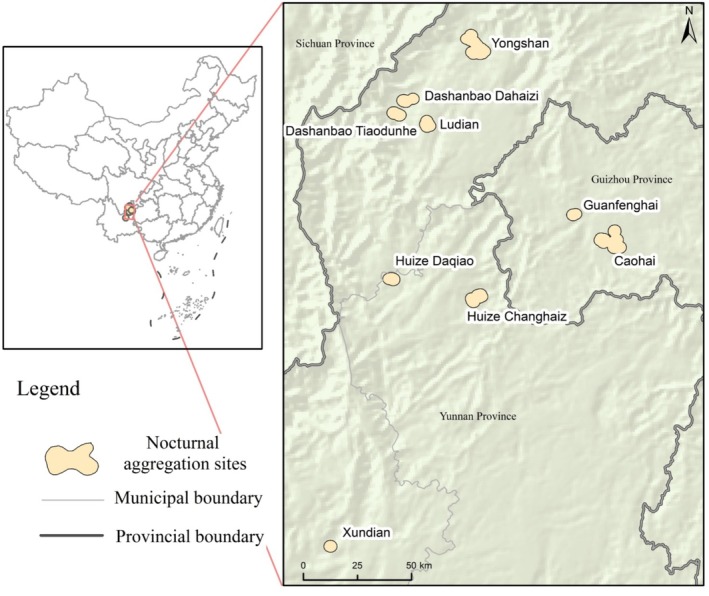
Study area: Nocturnal aggregation site of Black‐necked Crane eastern population wintering area.

The study was carried out in the wintering grounds of the eastern population of the Black‐necked Crane, with particular attention given to five nature reserves and their surrounding areas: Yongshan (103°15′ E‐104°01′ E, 27°31′ N‐28°32′ N), Dashanbao (103°14′ E‐103°23′ E, 27°18′ N‐27°28′ N), Huize (comprising the Daqiao Reservoir and Changhaizi Reservoir) (103°03′ E‐103°55′ E, 25°48′ N‐27°04′ N), Caohai (104°12′ E‐104°18′ E, 26°49′ N‐26°53′ N), and Xundian (102°41′ E‐103°33′ E, 25°20′ N‐26°01′ N) (Figure 1). These wintering sites are primarily distributed across the central and northern regions of the Yunnan‐Guizhou Plateau, situated between the Wumeng and Wulian mountain ranges, with elevations ranging from 2200 to 3400 m. The region is characterized by a subtropical plateau monsoon climate; the average temperature in January ranges from −1°C to 2°C, and the annual precipitation is approximately 1000 mm (Li et al. 2005). It is noteworthy that the wetland resources in Yongshan and Xundian are relatively scarce and the marshes have severely degraded, while in several other areas, the presence of reservoirs or natural lakes has resulted in comparatively rich wetland resources (Li et al. 2005; Lu and Yang 2014; Wang et al. 2014). According to the National Bureau of Statistics of China (2021), Yongshan County, Zhaotong City, Yunnan Province, China, has approximately 30,000 residents (encompassing Maolin Town and Wuzhai Township), while Dashanbao Town, Zhaotong City, Yunnan Province, China, has around 10,000 residents (Dashanbao Town). Huize County, Qujing City, Yunnan Province, China, which includes Daqiao Township, Zhehai Town, and Kuangshan Town, is home to approximately 110,000 residents. Caohai Town, Bijie City, Guizhou Province, China, consisting of Caohai Town and Shuanglong Town, has roughly 60,000 residents, and Liushao Township, Xundian County, Kunming City, Yunnan Province, China, accounts for around 20,000 residents. Owing to its high‐altitude, cold‐climate environment, the region exhibits a fragile economic foundation and a limited industrial structure, with agriculture and animal husbandry serving as the predominant economic pillars.

### 2.2 Satellite Tracking of the Black‐necked Cranes

From 2015 to 2022, we set hundreds of snap nooses on the lakeshore near crane resting sites. Each snap set contained nearly 100 nooses, with nooses set perpendicular to the lakeshore and secured with two large sticks. A capture team remained out of sight about 500 m away, with a good view of the trap site, and quickly (within 1 min) restrained cranes caught in a noose. Subsequently, we promptly attached GPS (Global Positioning System) transmitters to the upper tarsi of the cranes' left legs and affixed red color bands with white inscriptions to their right legs. The entire procedure was completed within 10 min. Among the study individuals, a subset comprised Black‐necked Cranes rescued by the reserve. Upon meeting the criteria for reintroduction to the wild, these individuals were also equipped with GPS transmitters and color band markers (Table 1). Notably, all released Black‐necked Cranes exhibited no abnormal behaviors post‐release.

**TABLE 1:** Information on tracked Black‐necked Cranes of the eastern wintering population.

Individual	Capture day	Data start time	Data end time	Track days	Location of capture	Status	Method	Minimum time interval
BNC 01	2015‐02‐14	2016‐05‐05	2017‐05‐05	366	Dashanbao	Juvenile	Leg trap	1 h
BNC 02	2015‐03‐17	2016‐05‐05	2017‐05‐02	360	Dashanbao	Adult	Leg trap	1 h
BNC 03	2015‐03‐20	2016‐05‐05	2017‐05‐04	357	Dashanbao	Adult	Leg trap	1 h
BNC 04	2016‐01‐05	2016‐01‐05	2016‐04‐03	89	Yongshan	Juvenile	Rescue	1 h
BNC 05	2016‐01‐05	2016‐01‐05	2016‐03‐09	22	Yongshan	Juvenile	Rescue	1 h
BNC 06	2018‐01‐17	2018‐01‐17	2018‐03‐29	72	Daqiao	Adult	Leg trap	1 h
BNC 07	2019‐01‐06	2019‐01‐06	2019‐06‐26	125	Caohai	First year	Leg trap	1 h
BNC 08	2020‐12‐02	2020‐12‐16	2021‐06‐10	177	Daqiao	Juvenile	Leg trap	1 h
BNC 09	2021‐03‐18	2021‐03‐18	2022‐09‐15	446	Daqiao	First year	Leg trap	1 h
BNC 10	2021‐03‐18	2021‐03‐19	2023‐12‐11	998	Daqiao	First year	Leg trap	1 h
BNC 11	2021‐03‐18	2021‐03‐19	2022‐03‐21	369	Daqiao	First year	Leg trap	1 h
BNC 12	2021‐11‐27	2021‐11‐27	2022‐03‐19	112	Dashanbao	Juvenile	Rescue	1 h
BNC 13	2022‐02‐27	2022‐02‐27	2023‐12‐11	653	Changhaizi	Adult	Leg trap	15 min
BNC 14	2022‐03‐02	2022‐03‐03	2023‐12‐11	649	Xundian	First year	Leg trap	15 min

The satellite trackers used were HQLG4021S model (Hunan Global Messenger Technology Co., China). The total weight of the satellite tracker was 44 g, which accounted for < 3% of the crane's weight and did not impede the crane's normal activities (Barron et al. 2010). These solar‐powered devices use GPS technology to record data, including tracker ID, date, time, latitude and longitude coordinates, altitude, and positioning accuracy. These data were transmitted back through the GSM (Global System for Mobile Communications) network. To further minimize errors caused by the trackers' positioning, this study only retained data points with a vertical error of < 200 m (Wen et al. 2022), totaling 21,683 points.

### 2.3 Identifying Clusters of Nocturnal Roosting Sites

The nocturnal roosting points were selected from valid points between 22:00 and 6:00 the next day (Kong et al. 2008), ensuring at least four valid positioning points each night (The monthly data for the Black‐necked Crane is shown in Table S1). These night roosting points were distributed across six counties in two provinces (Yunnan and Guizhou). To avoid erroneous inferences that might arise from combining data from different populations (Alldredge and Griswold 2006), we identified distinct nocturnal aggregation sites for the eastern population of Black‐necked Cranes. Notably, we did not use protected areas as proxies for clusters of nocturnal roosting sites because some protected areas encompass multiple large wetlands, and the significant distances between these wetlands make such an approach unsuitable.

During the wintering period, Black‐necked Cranes exhibit flocking behavior, with group sizes ranging from a few individuals to several dozen. Additionally, their specific nocturnal roosting locations within these aggregation sites are not fixed; they may roost either at the center or the edges of the sites. To account for this spatial variability, we used a kernel density model to analyze the night roosting points (Powell 2000). This approach allowed us to delineate the nocturnal aggregation sites of the eastern population based on the cranes' nocturnal movement patterns.

Specifically, we applied the kernel density model to each individual nocturnal roosting point, retained the 50% kernel density to identify the core areas of aggregation, and established a 2 km buffer zone around these core areas to define the boundaries of the nocturnal aggregation sites. This buffer distance was chosen based on the typical nocturnal movement range of Black‐necked Cranes, ensuring that the delineated sites accurately reflected their spatial distribution and behavior. Nine clusters of nocturnal roosting sites were identified, and a 2 km buffer zone of each cluster was called a nocturnal aggregation site (Figure 1).

### 2.4 Type of Nocturnal Roosting Points

Indexing the 2021 global 10 m resolution land cover dataset (Karra et al. 2021) (Esri | Sentinel‐2 Land Cover Explorer), we obtained the categories of Black‐necked Crane night roosting points, mainly including two categories: shallow water nocturnal roosting point and terrestrial nocturnal roosting point. To further ensure the accuracy of the Black‐necked Crane night roosting point categories, we corrected all land roosting points with Sentinel‐2 satellite imagery from adjacent time periods (±1 month) (https://browser.dataspace.copernicus.eu/) to further guarantee the accuracy of the results.

### 2.5 Factors Related to Terrestrial Nocturnal Roosting Site Selection of Black‐necked Cranes

#### 2.5.1 Area of Water Body Within Each Nocturnal Aggregation Site

We used Google Earth Engine (GEE, https://earthengine.google.com/) to calculate the NDWI (Normalized Difference Water Index) from Sentinel imagery (since the annual change in water body area was not significant, we selected imagery from November 2022 for extraction) to obtain the area of open water within each nocturnal aggregation site.

#### 2.5.2 Terrain Factors

The Topographic Position Index (TPI) quantifies the elevation difference between a focal point and the weighted average elevation of its surrounding area (Fels and Zobel 1995). This metric is commonly used to assess whether a given location is situated higher or lower relative to its surroundings. A positive value indicates that the target location is higher than its surroundings, whereas a negative value indicates it is lower. We calculated the weighted average elevation of a 12.5 m DEM (Digital Elevation Model) (ALOS PALSAR, ASF Data Search) within 5 × 5 cell and 21 × 21 cell windows. Referring to Weiss's (2001) classification method and Lu's (2018) summary of Black‐necked Crane land roosting sites, we categorized the terrain into three types: highland, lowland, and valley (Table 2). In addition to TPI, we also considered the effects of elevation, slope, southward (*S = −cos*(aspect*π/180), −1 to 1. 1 represents due south, while −1 denotes due north.), and eastward (*E = sin*(aspect*π/180), −1 to 1. 1 represents due east, while −1 denotes due west.) on the Black‐necked Crane's selection of terrestrial nocturnal roost sites. All of these factors were computed using a 12.5 m DEM. We randomly selected 5000 points within a 1 km buffer zone around the night roosting points location as control points.

**TABLE 2:** Descriptions of landform classes and reclass landform classification.

Landform classification (Weiss 2001)	TPI criteria		Reclass landform classification
	Small neighborhood * 5 cell	Large neighborhood 21 * 21 cell	
Upland drainages, Headwaters	TPI ≤ −1 SD	TPI ≥ 1 SD	Highland
Upper slopes, mesas	−1 SD < TPI < 1SD	TPI ≥ 1 SD	Highland
Local ridges, hills in valleys	TPI ≥ 1 SD	TPI ≤ −1 SD	Highland
Mountain tops, high ridges	TPI ≥ 1 SD	TPI ≥ 1 SD	Highland
Plains(slope < 5°)	−1 SD < TPI < 1SD	−1 SD < TPI < 1SD	Lowland
Open slopes (slope > 5°)	−1 SD < TPI < 1SD	−1 SD < TPI < 1SD	Lowland
Mid‐slope ridges, small hills in plains	TPI ≥ 1 SD	−1 SD < TPI < 1SD	Lowland
Canyons, deeply incised streams	TPI ≤ −1 SD	TPI ≤ −1 SD	Valley
Mid‐slope drainages, Shallow valleys	TPI ≤ −1 SD	−1 SD < TPI < 1SD	Valley
U‐shaped valleys	−1 SD < TPI < 1SD	TPI ≤ −1 SD	Valley

#### 2.5.3 Anthropogenic Disturbance Factors

We considered the distance from roads (≥ 2 m in width) and the distance from buildings as anthropogenic disturbance factors influencing terrestrial nocturnal roosting of Black‐necked Cranes. First, we delineated roads and residential areas in Google Earth, and then calculated the straight‐line distances from these features to both the Black‐necked Crane terrestrial roosting points and the control points.

### 2.6 Levels of Nocturnal Activity in Different Types of Nocturnal Roosting Sites

Diurnal birds are often in a stable state at night, but when disturbances occur around the nocturnal roosting site, Black‐necked Cranes may move. Therefore, we calculated the distances between all roosting points of an individual during that night and used the maximum distance as the night movement distance of the Black‐necked Crane for that night. By comparing the movement distance of two roosting types, we explored the nocturnal activity levels of Black‐necked Cranes in shallow water environments and on land. We classified nocturnal movement distances into two categories (≤ 500 m vs. > 500 m) and used the chi‐squared test to assess whether Black‐necked Cranes exhibit different nocturnal activity levels (movement distance) among various roosting site types.

### 2.7 Data Analysis

To compare the selection of different terrains by Black‐necked Cranes, we used the chi‐square test along with Vanderploeg selectivity coefficients (*W*_*i*_) and the Scavia selectivity index (*E*_*i*_) (Vanderploeg and Scavia 1979) to calculate the preference of Black‐necked Cranes for different terrains. The formulas are as follows:

$$W_{i}=\left(r_{i}/p_{i}\right)/\Sigma \;\left(r_{i}/p_{i}\right)$$

$$E_{i}=\left(w_{i}-\frac{1}{n}\right)/\left(w_{i}+\frac{1}{n}\right)$$

In the formulas, *n* represents the number of classification levels, which in this study is *n* = 3; *r*_*i*_ indicates the proportion of Black‐necked Cranes appearing in a certain terrain category; *p*_*i*_ is the proportion of that category in the total. *E*_*i*_ ranges from −1 to 1, with values closer to −1 indicating avoidance, closer to 1 indicating preference, and *E*_*i*_ equal to or near 0 indicating random selection.

All statistical calculations were performed using R v 4.4.1 (R Core Team 2024), with the following packages used: geosphere (Hijmans et al. 2017), raster (Hijmans et al. 2015), and sf (Pebesma and Bivand 2023).

## 3 Results

### 3.1 Proportion and Influencing Environmental Factors of Different Types of Nocturnal Roosting Sites in Black‐necked Cranes

**FIGURE 2:**
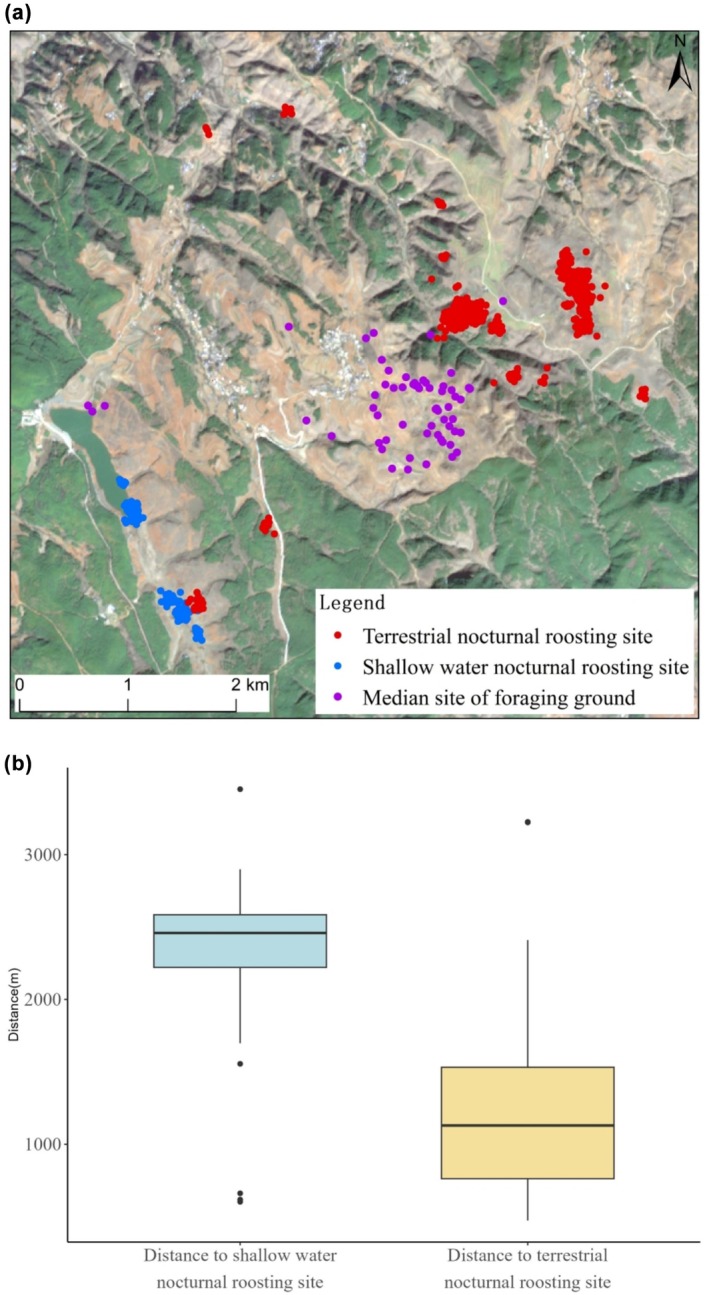
(a) Distribution of nocturnal roosting sites and foraging points within the Changhaizi Reservoir area. (b) Comparison of the distance from Black‐necked Crane median site of foraging ground to the nearest shallow water nocturnal roosting site and the nearest terrestrial nocturnal roosting site.

Most nights, Black‐necked Cranes roost in shallow water (1182 nights, accounting for 78.2% of the total roosting nights). They also roost on land (301 nights, accounting for 19.9% of the total roosting nights) or mixed nocturnal roosting sites (29 nights, accounting for 1.9% of the total roosting nights). The larger the area of open water, the lower the frequency of Black‐necked Cranes utilizing terrestrial nocturnal roosting sites. However, the personality of the Black‐necked Crane (BNC 06 and BNC 07) will influence this trend (Table 3 and Table 4, the influence mechanism of specific individuals on the trend of the terrestrial roosting sites ratio in the Tiaodunhe and Caohai area is explained in detail in the discussion). Additionally, in areas where Black‐necked Cranes choose land roosting sites (95 nights) and shallow water roosting sites (38 nights) (Changhaizi in Huize), Black‐necked Cranes typically select land roosting sites closer to their foraging grounds as their overnight locations (Figure 2).

**TABLE 3:** Water area, terrestrial nocturnal roosting site proportion, and number of nights by location.

Location	Water body area within the nocturnal aggregation sites (ha)	Proportion of terrestrial roosting site (%)	Number of nigths
Yongshan	4.9	100	72
Ludian	0.2	100	70
Xundian	0.8	100	13
Guanfenghai	0.9	75	16
Huize Changhaiz	20.4	63	149
Dashanbao Dahaizi	34.5	26.2	229
Dashanbao Tiaodunhe	249.9	18	83
Caohai	1783.3	4.6	411
Huize Daqiao	447.6	0	469

**TABLE 4:** Proportion of different nocturnal roosting site types used by Black‐necked Cranes (yellow: terrestrial nocturnal roosting site, blue: shallow water nocturnal roosting site, gray: mixed nocturnal roosting site). Only data were retained for individual Black‐necked Cranes staying more than 10 days at a single nocturnal aggregation site.

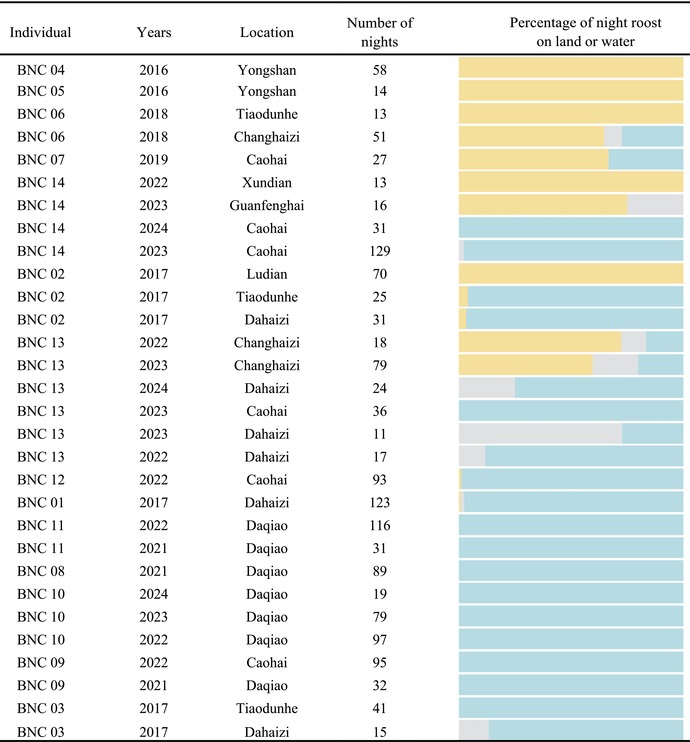

### 3.2 Topographic Characteristics of the Terrestrial Nocturnal Roosting Sites of Black‐necked Crane

In three completely terrestrial nocturnal aggregation sites, Black‐necked Cranes show significant differences in their utilization of highland terrain (Yongshan: *p* = 0.045; Ludian: *p* < 0.001; Xundian: *p* < 0.001). The index selection results indicate that Black‐necked Cranes prefer to roost in highland terrain (headwaters, uphill terraces, mountain tops, and local ridges) (Yongshan: *E*_*i*_ = 0.083; Ludian: *E*_*i*_ = 0.274; Xundian: *E_i_* = 0.316), avoid valley terrain (canyons, shallow valleys, and U‐shaped valleys) (Yongshan: *E*_*i*_ = −0.073; Ludian: *E*_*i*_ = −0.448; Xundian 9: *E*_*i*_ = −0.365), and use lowland terrain (open slopes, plain hills, and plains) under a strategy characterized by randomness and avoidance (Yongshan: *E*_*i*_ = −0.022; Ludian: *E*_*i*_ = −0.071; Xundian: *E*_*i*_ = −0.243) (Table 5). Due to variations in the regions inhabited by Black‐necked Cranes, they exhibit different preferences for elevation, slope, distance from roads, distance from buildings, and southward and eastward (see Figure S1).

**TABLE 5:** Topographic selection characteristics of entirely terrestrial nocturnal roosting congregations for Black‐necked Cranes.

Location	Landform	*χ* ^2^	*p*	*W*_*i*_	*E*_*i*_	No. of roosts	No. of control
Yongshan	**Highland**	**4.032**	**0.045**	**0.394**	**0.083**	**67**	**840**
	Lowland	0.017	0.895	0.319	−0.022	101	1566
	Valley	2.639	0.104	0.288	−0.073	151	2594
Ludian	**Highland**	**227.385**	**< 0.001**	**0.585**	**0.274**	**274**	**907**
	Lowland	3.326	0.068	0.289	−0.071	241	1616
	**Valley**	**177.638**	**< 0.001**	**0.127**	**−0.448**	**162**	**2477**
Xundian	**Highland**	**166.817**	**< 0.001**	**0.641**	**0.316**	**133**	**697**
	Lowland	2.752	0.097	0.203	−0.243	86	1421
	**Valley**	**56.838**	**< 0.001**	**0.155**	**−0.365**	**133**	**2882**

*Note:* No. of roosts: Number of nocturnal roosting points, No. of control: Number of control points. Bold items indicate that Black‐necked Cranes significantly select this landform.

### 3.3 Nocturnal Movement Status of Black‐necked Cranes

**FIGURE 3:**
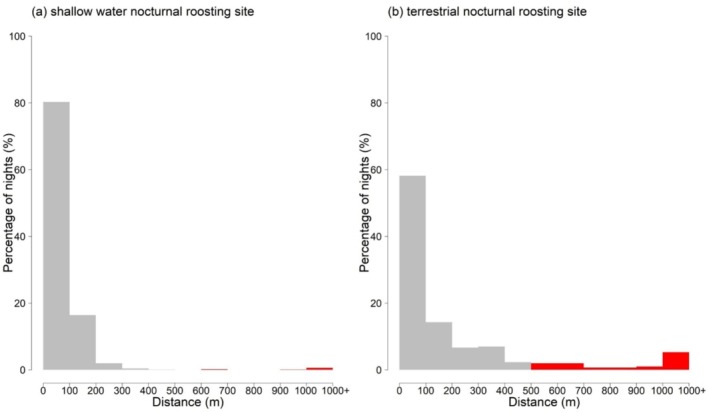
Proportion of night‐time movements of Black‐necked Cranes in two types of nocturnal roosting sites: (a) shallow water nocturnal roosting site; (b) terrestrial nocturnal roosting site. Red fill indicates night‐time movements greater than 500 m.

The eastern population of Black‐necked Cranes exhibits greater stability when roosting in shallow water, with a significantly lower probability of nocturnal movements exceeding 500 m compared to roosting on land (11.6% vs. 0.8%, *χ* ^2^ = 91.157, df = 1, *p* < 0.001) (Figure 3).

## 4 Discussion

### 4.1 Water Body Area, Foraging Ground Location, and Individual Personality Influenced the Proportion of Terrestrial Nocturnal Roosting in Black‐necked Cranes

To cope with environmental pressures, animals make appropriate adjustments. In this study, we obtained the selection ratios of nocturnal roosting site types during the night. The choices of roosting sites by Black‐necked Cranes varied across different nocturnal aggregation sites. In wintering sites with shallow water, Black‐necked Cranes predominantly choose to roost in shallow water. However, in areas lacking shallow water, their roosting sites shift to terrestrial locations. Similar phenomena have been observed in studies of Sandhill Cranes ( *Grus canadensis* ) (Krapu et al. 1984). Our results show that Black‐necked Cranes do not exclusively depend on aquatic environments during nocturnal roosting. Instead, they exhibit flexible nocturnal roosting site selection based on the surrounding environment. When the extent of the water body surrounding the nocturnal roosting site is sufficiently large, opting for shallow water as a roosting location is a strategic and ecologically sound decision. However, when the water body area is too small to form an effective barrier, the cranes shift their nocturnal roosting sites to mountainous areas.

The location of foraging grounds influences the selection of nocturnal roosting site types by Black‐necked Cranes. We found that Black‐necked Cranes wintering at Changhaizi sometimes chose to roost in shallow water and sometimes on land, with the proportion of terrestrial nocturnal roosting being greater than that of shallow water. Lu (2018) obtained the trend of the Black‐necked Crane population's shift in nocturnal roosting points types at Changhaizi, noting a trend from shallow water to terrestrial nocturnal roosting with the extension of wintering time. Due to the limitations of field observation, individual identification was not possible, and thus, the trend of roosting sites utilization at the individual level could not be tracked in the past. Our study revealed that at the individual level, the selection of nocturnal roosting sites types by Black‐necked Cranes is random and uncertain, but in terms of collective level, Black‐necked Cranes at Changhaizi prefer terrestrial roosting sites. During the overwintering period, the diet of the Black‐necked Crane primarily consists of plant‐based food sources, supplemented by the consumption of insects and fish. The plant‐based diet primarily comprises Cyperaceae species, potato ( *Solanum tuberosum* ), and corn ( *Zea mays* ), while the animal‐based diet includes fish and common earthworm ( *Lumbricus terrestris* ) (Li 1997; Hu et al. 2002; Bishop and Li 2002). Therefore, fallow farmland is one of the important feeding grounds for the Black‐necked Crane. We consider that the Black‐necked Crane' preference for terrestrial roosting at Changhaizi is related to the proximity of these sites to fallow farmland, as a shorter distance can conserve energy. Other studies have also shown that cranes' feeding grounds are not too far from their roosting sites (Yoo et al. 2011).

Additionally, among the studied individuals, we observed two individuals (BNC 06 and BNC 07) exhibiting different nocturnal roosting selection. Both individuals showed a selection for terrestrial roosting, despite the availability of sufficiently large water bodies at Dashanbao Tiaodunhe and Caohai. Whether this behavior is associated with individual‐specific traits, population isolation, or other factors remains unclear and warrants further investigation.

### 4.2 The Highland Terrain Is a Key Feature of the Black‐necked Crane's Terrestrial Roosting Sites

Safety is one of the primary characteristics of nocturnal roosting sites (Elmore et al. 2004). It is noteworthy that among the nine nocturnal roosting sites, only three (Yongshan, Ludian, and Xundian) were exclusively terrestrial. These roosting sites were primarily distributed on ridges or platforms on slopes (see Figure S2). Notably, Black‐necked Cranes exhibited significant and marginally significant (Limitations of the TPI calculation window) avoidance for lowland and valley. These patterns likely reflect the lack of critical safety features in such habitats: valley topography may restrict visibility and escape routes, while lowland zones could expose cranes to higher predation risks. Ridges or platforms on slopes not only provide relative safety but also offer an open view.

Overall, the terrestrial roosting sites of Black‐necked Cranes are mostly located in areas with steep overall slopes but locally flat and safe places, such as terraced fields after reclamation, platforms near mountain tops, and small paths on ridges. This avoidance of valleys and lowlands further reinforces the cranes' prioritization of security over accessibility in nocturnal roosting site selection. These areas can effectively avoid disturbances, facilitate the cranes' rest at night, and allow for emergency take‐off using the terrain features of the slope in case of sudden events. Studies on Sandhill Cranes have also found that they choose areas far from the shore as roosting sites, using water as a safety barrier (Krapu et al. 1984). As a medium to large bird, Black‐necked Cranes require a certain distance for take‐off and landing, which locally flat terraced fields can provide (Lu 2014). Moreover, flat areas can serve as secondary resting places to attract companions (Krapu et al. 2011).

We conducted a real‐time analysis of the elevation and aspect of the Black‐necked Crane's terrestrial roosting sites in relation to the daily meteorological data from the observation stations. The results indicate no significant correlation between these topographical features and weather factors (see Supporting Informations: Pages 4–5). As a species that inhabits the plateau year‐round, Black‐necked Cranes can physiologically endure lower temperatures than Gray Cranes ( *Grus grus* ) (Kong et al. 2016). This physiological adaptation may explain their relative insensitivity to meteorological conditions when selecting roosting locations at night. Nevertheless, compared to exposed shallow water roosting sites, Black‐necked Cranes may prefer topographical features that provide some degree of shelter during terrestrial roosting, such as leeward slopes. However, the existing meteorological station (Zhaotong Station) is located approximately 30 km from the nearest Black‐necked Crane nocturnal aggregation site (Ludian), with an elevation difference of approximately 500–600 m between Zhaotong Station (1910 m) and the Ludian nocturnal aggregation site(mean elevation: 2400 m), resulting in differing meteorological conditions compared to the nocturnal roosting sites. As such, the current study cannot clearly define the relationship between weather conditions and the slope aspect of the terrestrial roosting site. We plan to address this in future studies by installing micro‐meteorological stations in Black‐necked Crane terrestrial roosting areas to quantify the dynamic relationship between meteorological parameters and roosting behavior.

Black‐necked Cranes do not necessarily benefit from increased distance to roads or residential areas when selecting terrestrial nocturnal roost sites. At Yongshan, the roosting sites are situated only about 200 m from roads and 450 m from residential areas. However, high‐resolution imagery shows that rugged terrain significantly limits human access, creating a natural protective barrier. In contrast, the Black‐necked Cranes at Ludian (outside the protected area) and Xundian exhibit more concentrated nocturnal roosting sites that are farther from human disturbance factors. Nonetheless, there were 15 instances of nocturnal movements exceeding 500 m at Ludian and three such instances at Xundian. These observations suggest that rugged terrain can effectively shield Black‐necked Cranes from direct human interference.

### 4.3 Black‐necked Cranes That Roosted on Land at Night Are More Likely to Move

The nocturnal activity of diurnal birds is related to species, season, foraging, and human disturbance (Martin 2010; Gustin et al. 2014). Typically, Black‐necked Cranes do not engage in long‐distance nocturnal movements, but disturbances near their nocturnal roosting sites may force them to relocate. Statistical analysis in this study revealed that terrestrial nocturnal roosting Black‐necked Cranes exhibit higher nocturnal movement rates than those in shallow water. In Zhaotong Dashanbao, human and livestock disturbances are particularly intense during daytime, with documented cases of dogs attacking or killing cranes (Kong et al. 2008). Lu (2018) found three carcasses of Black‐necked Cranes at their terrestrial roosting sites (Changhaizi and Zhuanshanbao, the latter situated proximal to Ludian in Yunnan Province). All show signs of being preyed upon, but the exact cause of death could not be determined. In Bhutan, there have been records of Black‐necked Cranes being preyed upon by Leopards ( *Panthera pardus* ) (Choki et al. 2011), indicating that Black‐necked Cranes are prey for certain predators; the predator in this study area may be the Red Fox ( *Vulpes vulpes* ) (Wu et al. 2009). Our study suggests that when Black‐necked Cranes roost in shallow water at night, the surrounding water conditions make it difficult for free‐ranging dogs and wildlife to approach, resulting in relatively low disturbance intensity. However, when Black‐necked Cranes choose to roost on land at night, despite the terrain offering some protection, free‐ranging dogs and wildlife can approach more easily than in shallow water, thus increasing the disturbance intensity. When disturbances threaten the safety of Black‐necked Cranes, they are forced to fly away from the current site to find other safe nocturnal roosting sites.

## 5 Conservation Recommendations

Based on our research findings, we propose the following recommendations: (1) In the main wintering areas of the Black‐necked Crane, such as Yunnan Dashanbao Black‐necked Crane National Nature Reserve, Huize Black‐necked Crane National Nature Reserve, and Guizhou Caohai National Nature Reserve, it is crucial to protect wetland resources and prevent wetland degradation. (2) In areas where Black‐necked Cranes roost entirely on land, such as Yongshan and Xundian, efforts should be made to enhance the restoration and connectivity of small wetland resources. (3) Monitoring of the Black‐necked Cranes' terrestrial nocturnal roosting sites should be strengthened, such as by deploying infrared cameras and installing micro‐weather stations. (4) In all protected areas and their surrounding regions, the management of free‐ranging dogs should be strengthened.

## 6 Conclusion

From the analysis of our results, we can draw the following conclusions: (1) The eastern wintering population of Black‐necked Cranes selects different types of roosting sites in different regions, primarily determined by the area of the water body near the nocturnal roosting site, the location of their foraging grounds, and the personality of the crane. (2) Among various terrestrial roosting sites, Black‐necked Cranes prefer to roost in the highlands. (3) Black‐necked Cranes roosting on land are more likely to engage in nocturnal movements compared to those roosting in shallow water.

These findings contribute to the broader understanding of Black‐necked Crane wintering ecology, particularly in the context of nocturnal roosting site selection and nocturnal behavior. The use of GPS‐GSM tracking devices in this study represents a methodological advancement, enabling precise tracking and analysis of crane movements. This approach can be applied to other species to explore similar ecological questions.

## Author Contributions

**Xinlei Hou:** data curation (lead), formal analysis (lead), investigation (supporting), supervision (supporting), visualization (equal), writing – original draft (lead), writing – review and editing (equal). **Guangyi Lu:** conceptualization (supporting), writing – original draft (supporting), writing – review and editing (supporting). **Shuxia Zhang:** conceptualization (supporting), supervision (supporting), writing – original draft (supporting), writing – review and editing (supporting). **Liping Feng:** visualization (equal), writing – original draft (supporting). **Guopeng Ren:** conceptualization (lead), data curation (supporting), funding acquisition (equal), investigation (supporting), supervision (equal), visualization (supporting), writing – review and editing (equal). **Heqi Wu:** data curation (supporting), funding acquisition (equal), investigation (lead), resources (lead), supervision (lead), writing – original draft (supporting), writing – review and editing (supporting).

## Ethics Statement

This study was approved by the Institutional Animal Care and Use Committee, Kunming Institute of Zoology, Chinese Academy of Sciences (2021‐08‐004).

## Conflicts of Interest

The authors declare no conflicts of interest.

## Supporting information


Data S1

## Data Availability

All the required data are uploaded as Supporting Information.

## References

[ece371485-bib-0001] Alldredge, J. R. , and J. Griswold . 2006. “Design and Analysis of Resource Selection Studies for Categorical Resource Variables.” Journal of Wildlife Management 70: 337–346.

[ece371485-bib-0002] Barron, D. G. , J. D. Brawn , and P. J. Weatherhead . 2010. “Meta‐Analysis of Transmitter Effects on Avian Behaviour and Ecology.” Methods in Ecology and Evolution 1: 180–187. 10.1111/j.2041-210X.2010.00013.x.

[ece371485-bib-0003] Bishop, M. A. , and F. Li . 2002. “Effects of Farming Practices in Tibet on Wintering Black Necked Crane (*Grus nigricollis*) Diet and Food Availability.” Biodiversity Science 10: 393.

[ece371485-bib-0004] Boyce, M. S. , P. R. Vernier , S. E. Nielsen , and F. K. Schmiegelow . 2002. “Evaluating Resource Selection Functions.” Ecological Modelling 157: 281–300.

[ece371485-bib-0005] Chamberlain, M. J. , B. D. Leopold , and L. W. Burger . 2000. “Characteristics of Roost Sites of Adult Wild Turkey Females.” Journal of Wildlife Management 64: 1025–1032.

[ece371485-bib-0006] Chen, D. 1997. “Observations on the Wintering Behavior and Environmental Influences on Black‐Necked Cranes.” Chinese Biosphere Reserve 4: 11–17. (Chinese).

[ece371485-bib-0007] Choki, T. , J. Tshering , T. Norbu , U. Stenkewitz , and J. F. Kamler . 2011. “Predation by Leopards of Black‐Necked Cranes *Grus nigricollis* in Bhutan.” Forktail 27: 117–119.

[ece371485-bib-0008] del Hoyo, J. 1996. Handbook of the Birds of the World. 3. Hoatzin to Auks. Lynx Ed.

[ece371485-bib-0009] Elmore, L. W. , D. A. Miller , and F. J. Vilella . 2004. “Selection of Diurnal Roosts by Red Bats ( *Lasiurus borealis* ) in an Intensively Managed Pine Forest in Mississippi.” Forest Ecology and Management 199: 11–20. 10.1016/j.foreco.2004.03.045.

[ece371485-bib-0010] Engel, K. A. , and L. S. Young . 1992. “Movements and Habitat Use by Common Ravens From Roost Sites in Southwestern Idaho.” Journal of Wildlife Management 56: 596–602.

[ece371485-bib-0011] Fels, J. E. , and R. Zobel . 1995. “Landscape Position and Classified Landtype Mapping for Statewide DRASTIC Mapping Project.” North Carolina State University Technical Report VEL 95.

[ece371485-bib-0012] Gustin, M. , A. Ferrarini , G. Giglio , S. Pellegrino , and A. Frassanito . 2014. “First Evidence of Widespread Nocturnal Activity of Lesser Kestrel (*Falco naumanni*) in Southern Italy.” Ornis Fennica 91: 256–260. 10.51812/of.133862.

[ece371485-bib-0013] He, P. , D. Kong , Q. Liu , H. Yu , J. Zhao , and X. Yang . 2011. “Roosting‐Site Characteristics of Wintering Black‐Necked Cranes (*Grus nigricollis*) at Napahai, Yunnan.” Zoological Research 32, no. 2: 150–156 (Chinese). 21509960 10.3724/SP.J.1141.2011.02150

[ece371485-bib-0014] Hijmans, R. J. , J. Van Etten , J. Cheng , et al. 2015. “Package ‘raster’.” R package 734, 473.

[ece371485-bib-0015] Hijmans, R. J. , E. Williams , C. Vennes , and M. R. J. Hijmans . 2017. “Package ‘geosphere’.” Spherical trigonometry 1, 1–45.

[ece371485-bib-0016] Hu, J. , J. Wu , C. Dang , X. Zhong , and M. Dao . 2002. “Study on the Animal Foods of Wintering Black‐Necked Cranes (*Grus nigricollis*)at Dashanbao Reserve,Zhaotong,Yunnan.” Journal of Yunnan University: Natural Sciences Edition 24: 459–461.

[ece371485-bib-0017] IUCN . 2020. “*Grus nigricollis*: BirdLife International: The IUCN Red List of Threatened Species 2020: e.T22692162A180030167.” 10.2305/IUCN.UK.2020-3.RLTS.T22692162A180030167.en.

[ece371485-bib-0018] Jirinec, V. , C. P. Varian , C. J. Smith , and M. Leu . 2016. “Mismatch Between Diurnal Home Ranges and Roosting Areas in the Wood Thrush ( *Hylocichla mustelina* ): Possible Role of Habitat and Breeding Stage.” Auk 133: 1–12. 10.1642/AUK-15-76.1.

[ece371485-bib-0019] Johnsgard, P. A. 1983a. Cranes of the World [Complete Work]. Indiana University Press.

[ece371485-bib-0020] Johnsgard, P. A. 1983b. “Cranes of the World: Eurasian Crane (*Grus grus*).” In Cranes of the World, 227–237. Indiana University Press.

[ece371485-bib-0021] Karra, K. , C. Kontgis , Z. Statman‐Weil , J. C. Mazzariello , M. Mathis , and S. P. Brumby . 2021. “Global Land Use/Land Cover With Sentinel 2 and Deep Learning.” In: *2021 IEEE International Geoscience and Remote Sensing Symposium IGARSS*. IEEE, 4704–4707.

[ece371485-bib-0022] Kong, D. , F. Li , Q. Liu , X. Zhong , and X. Yang . 2016. “Flight of Black‐Necked Cranes ( *Grus nigricollis* ) With Legs Drawn Up: Behavioral Responses to Low Temperature and Duration of Exposure.” Wilson Journal of Ornithology 128: 144–149. 10.1676/wils-128-01-144-149.1.

[ece371485-bib-0023] Kong, D. , X. Yang , X. Zhong , M. Dao , and Y. Zhu . 2008. “Diurnal Time Budget and Behavior Rhythm of Wintering Black‐Necked Crane (*Grus nigricollis*) at Dashanbao in Yunnan.” Zoological Research 29: 195–202. (Chinese).

[ece371485-bib-0024] Krapu, G. L. , D. A. Brandt , K. L. Jones , and D. H. Johnson . 2011. “Geographic Distribution of the Mid‐Continent Population of Sandhill Cranes and Related Management Applications: Distribution Géographique de la Population des Grues du Canada Dans le Centre du Continent et Les Applications Relatives à Leur Gestion.” Wildlife Monographs 175: 1–38. 10.1002/wmon.1.

[ece371485-bib-0025] Krapu, G. L. , D. E. Facey , E. K. Fritzell , and D. H. Johnson . 1984. “Habitat Use by Migrant Sandhill Cranes in Nebraska.” Journal of Wildlife Management 48: 407–417.

[ece371485-bib-0026] Lashley, M. A. , M. V. Cove , M. C. Chitwood , et al. 2018. “Estimating Wildlife Activity Curves: Comparison of Methods and Sample Size.” Scientific Reports 8: 4173. 29520029 10.1038/s41598-018-22638-6 PMC5843653

[ece371485-bib-0027] Li, F. 2014. “IUCN Black‐Necked Crane (*Grus nigricollis*) Conservation Plan.” Zoological Research 35: 3–9.24470450

[ece371485-bib-0028] Li, F. , S. Fang , and Y. Guan . 2005. “Preliminary Survey on Wintering Environment of Black‐Necked Cranes in Northeast Yunnan.” In Status and Conservation of Black‐Necked Cranes on the Yunnan and Guizhou Plateau, edited by F. S. Li , X. J. Yang , and F. Yang , 76–92. Yunnan Nationalities Publishing House.

[ece371485-bib-0029] Li, L. 1997. “Population Ecology and Endangered Categories Evaluation of the Black‐Necked Crane (*Grus nigricollis*).” Chinese Biodiversity 5: 84–89. (Chinese).

[ece371485-bib-0030] Li, Z. , and F. Li . 2005. Study on Black‐Necked Cranes. Shanghai Technological and Educational Press.

[ece371485-bib-0031] Lu, G. 2014. “Roost on Mountain Slope: An Atypical Winter Nocturnal Roost Site Use by Black‐Necked Crane (*Grus nigricollis*) in Northeast Yunnan, China.” (Chinese).

[ece371485-bib-0032] Lu, G. 2018. “Upland Roost Sites Use by the Black‐Necked Crane (*Grus nigricollis*) in Northeast Yunnan.” (Chinese).

[ece371485-bib-0033] Lu, G. , R. Wang , L. Ma , and X. Yang . 2017. “Characteristics of Dry Upland Roosts of the Black‐Necked Crane ( *Grus nigricollis* ) Wintering in Yongshan, China.” Wilson Journal of Ornithology 129: 323–330. 10.1676/15-191.1.

[ece371485-bib-0034] Lu, G.‐Y. , and X.‐J. Yang . 2014. “Black‐Necked Cranes (*Grus nigricollis*) Wintering in Yongshan County, Yunnan and Their Conservation.” Zoological Research 35: 143–150. 10.3724/SP.J.1141.2011.02150 21509960

[ece371485-bib-0035] Martin, G. 2010. Birds by Night. A&C Black.

[ece371485-bib-0036] Masse, R. 2014. “American Woodcock Ecology and Bird Conservation in Relation to Forest Management.” Kingston, RI. 10.23860/diss-masse-roger-2014.

[ece371485-bib-0037] Meine, C. , and G. Archibald . 1996. The Cranes: Status Survey and Conservation Action Plan. IUcN.

[ece371485-bib-0038] National Bureau of Statistics of China . 2021. Tabulation on the 2020 Population Census of the People's Republic of China by Township. China Statistics Press.

[ece371485-bib-0039] Ordiz, A. , S. Saebø , J. Kindberg , J. E. Swenson , and O.‐G. Støen . 2017. “Seasonality and Human Disturbance Alter Brown Bear Activity Patterns: Implications for Circumpolar Carnivore Conservation?” Animal Conservation 20: 51–60. 10.1111/acv.12284.

[ece371485-bib-0040] Pebesma, E. , and R. Bivand . 2023. Spatial Data Science: With Applications in R. Chapman and Hall/CRC.

[ece371485-bib-0041] Peng, M. , Z. Wang , and X. Zhong . 2013. Comprehensive Scientific Investigation of Black‐Necked Crane Nature Reserve in Dashanbao, Yunnan. Science Press. (Chinese).

[ece371485-bib-0042] Powell, R. 2000. “Animal Home Ranges and Territories and Home Range Estimators.” In Research Techniques in Animal Ecology: Controversies and Consequences, 65–110. Columbia University Press.

[ece371485-bib-0043] Qiu, G. , G. Huang , and F. Liu . 1995. “Ecological Studies and Conservation of Wintering Black‐Necked Cranes in Eastern and Northeastern Yunnan.” Environmental Science Survey 24: 40–43. (Chinese).

[ece371485-bib-0044] R Core Team . 2024. R: A Language and Environment for Statistical Computing. R Foundation for Statistical Computing.

[ece371485-bib-0045] Tsamchu, D. , D. Gongsang , F. Li , and L. Yang . 2014. “Roost Sites of Black‐Necked Cranes(*Grus nigricollis*)wintering at Shigaze Area in the Yarlung Zangbo Valley of Tibet, China.” Zoological Research 35: 205–207. (Chinese).

[ece371485-bib-0046] Vanderploeg, H. A. , and D. Scavia . 1979. “Calculation and Use of Selectivity Coefficients of Feeding: Zooplankton Grazing.” Ecological Modelling 7: 135–149. 10.1016/0304-3800(79)90004-8.

[ece371485-bib-0047] Wang, R.‐X. , W.‐X. Luo , D.‐J. Kong , et al. 2014. “Changes in Number and Location of Black‐Necked Cranes (*Grus nigricollis*) Wintering at Xundian, Yunnan Province.” Zoological Research 35: 151–257.

[ece371485-bib-0048] Weiss, A. D. 2001. “Topographic Position and Landforms Analysis.” Poster Presentation, ESRI User Conference, San Diego, 9–13 July 2001.

[ece371485-bib-0049] Wen, H. , G. Ren , and Z. Huang . 2022. “Performance Assessment of GPS Collar Over Mountainous Region.” (Chinese).

[ece371485-bib-0050] Wu, H. , K. Zha , M. Zhang , and X. Yang . 2009. “Nest Site Selection by Black‐Necked Crane *Grus nigricollis* in the Ruoergai Wetland, China.” Bird Conservation International 19: 277–286. 10.1017/S0959270909008168.

[ece371485-bib-0051] Wu, Z. , Z. Li , Y. Wang , and Y. Jiang . 1993. “Migration of Black‐Necked Crane in China.” Acta Zoologica Sinica 39: 105–106. (Chinese).

[ece371485-bib-0052] Wu, Z. , K. Zhang , W. Li , and P. Jiang . 2013. “Number, Habitats, and Roosting Sites of Wintering Black‐Necked Cranes in Huize Nature Reserve, Yunnan, China.” Mountain Research and Development 33: 314–322. 10.1659/MRD-JOURNAL-D-11-00066.1.

[ece371485-bib-0053] Yoo, S.‐H. , H.‐S. Kwon , J.‐J. Park , and C.‐H. Park . 2011. “Spatial Distribution of Feeding Site and the Relationship Between Density and Environmental Factors (Roosting Site, Road and Residence) of Cranes in Cheorwon Basin, Korea.” Korean Journal of Environment and Ecology 25: 516–525.

