# Supplementary material for: Terrestrial Nocturnal Roosting Behavior of Black‐necked Cranes (Grus nigricollis) on the Yunnan‐Guizhou Plateau: Active Choice or Forced Environmental Adaptation
Source: Ecol Evol. 2025 Jun 13;15(6):e71485. doi: 10.1002/ece3.71485 (PMC12165949; doi:10.1002/ece3.71485)
Supplement: Supplementary file 1 — Data S1 [file ECE3-15-e71485-s001.docx]

**Supplementary materials**

Figure S1 Environmental characteristics of nocturnal roosting points vs. control points. (a: Yongshan, b: Ludian, c: Xundian)


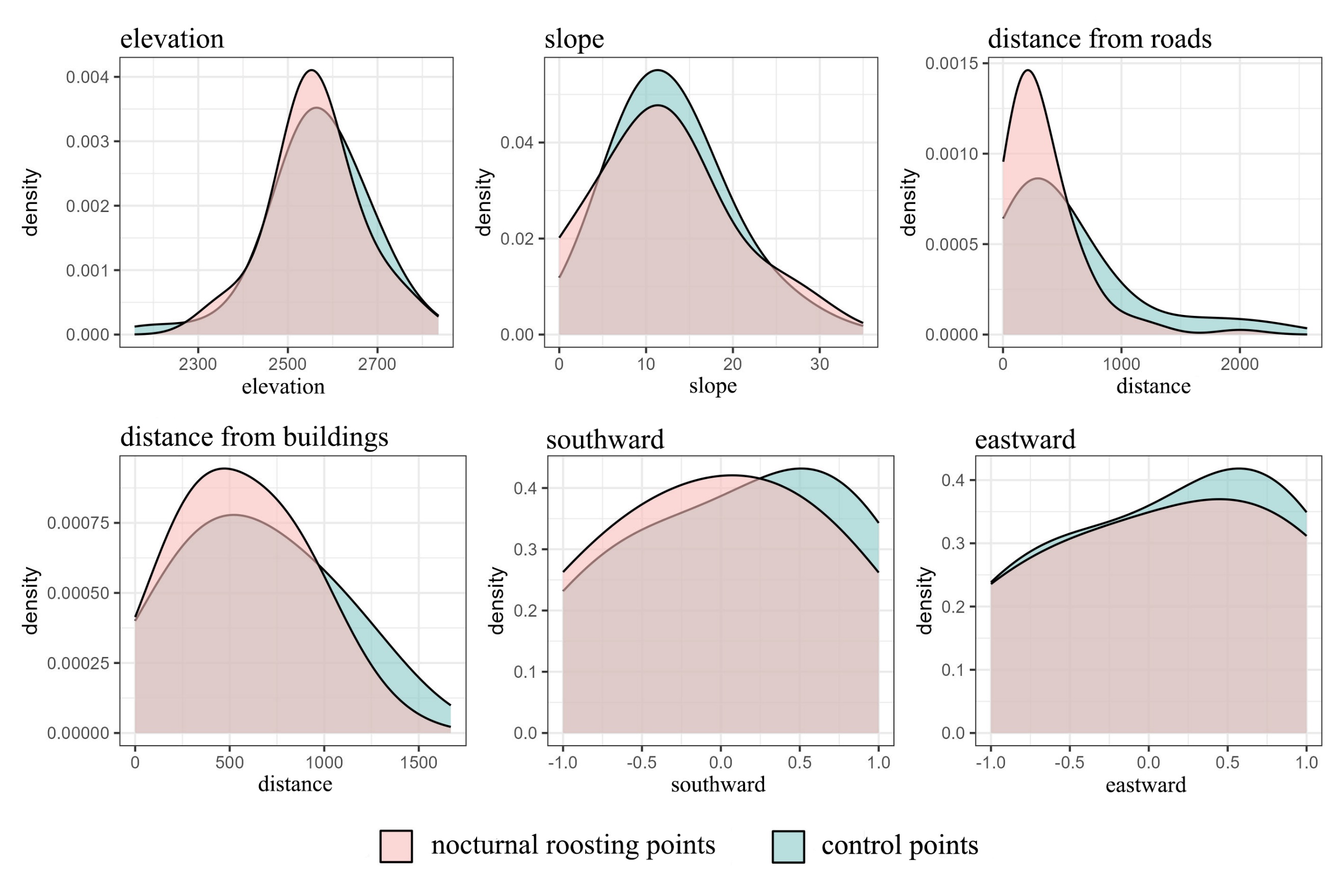

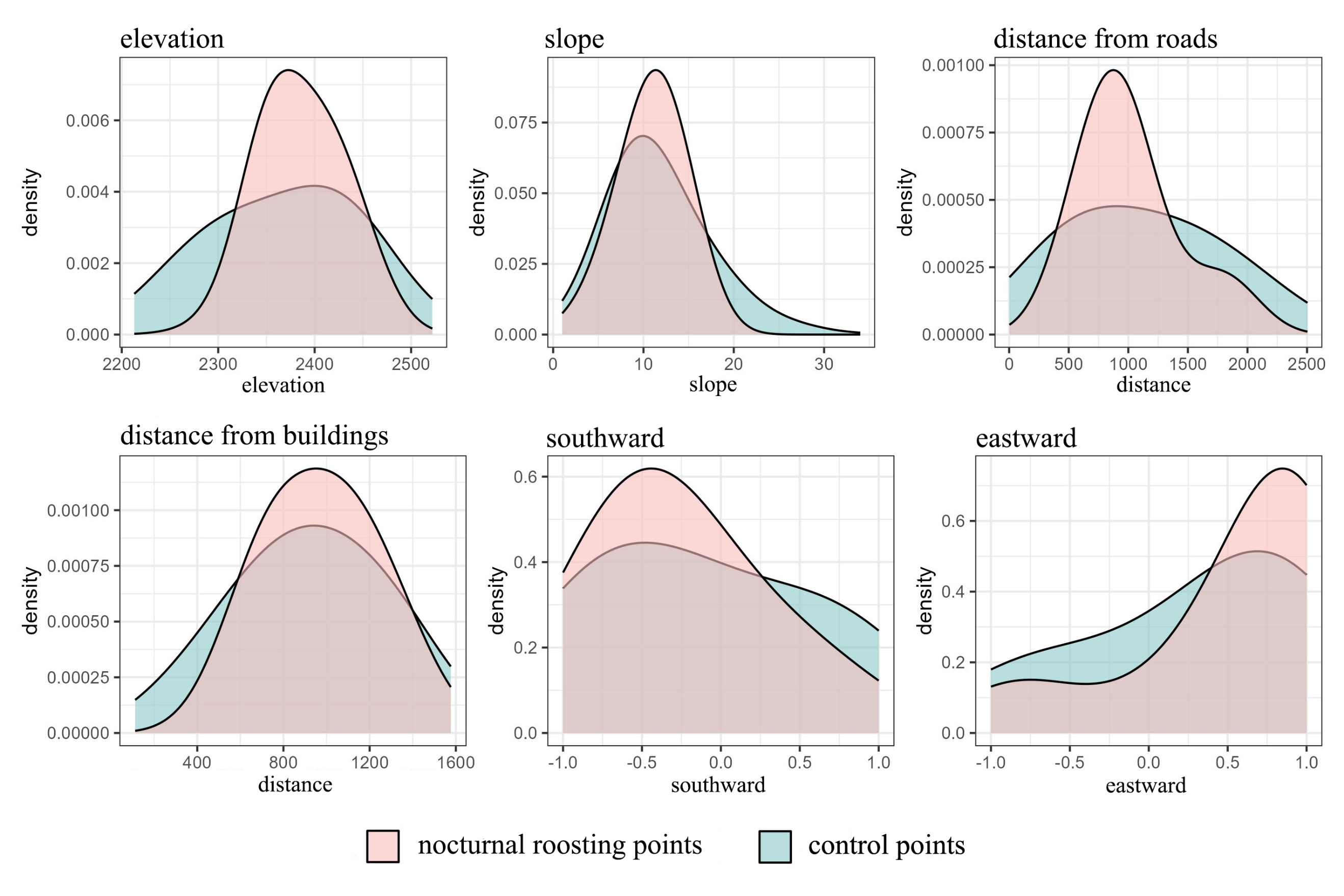

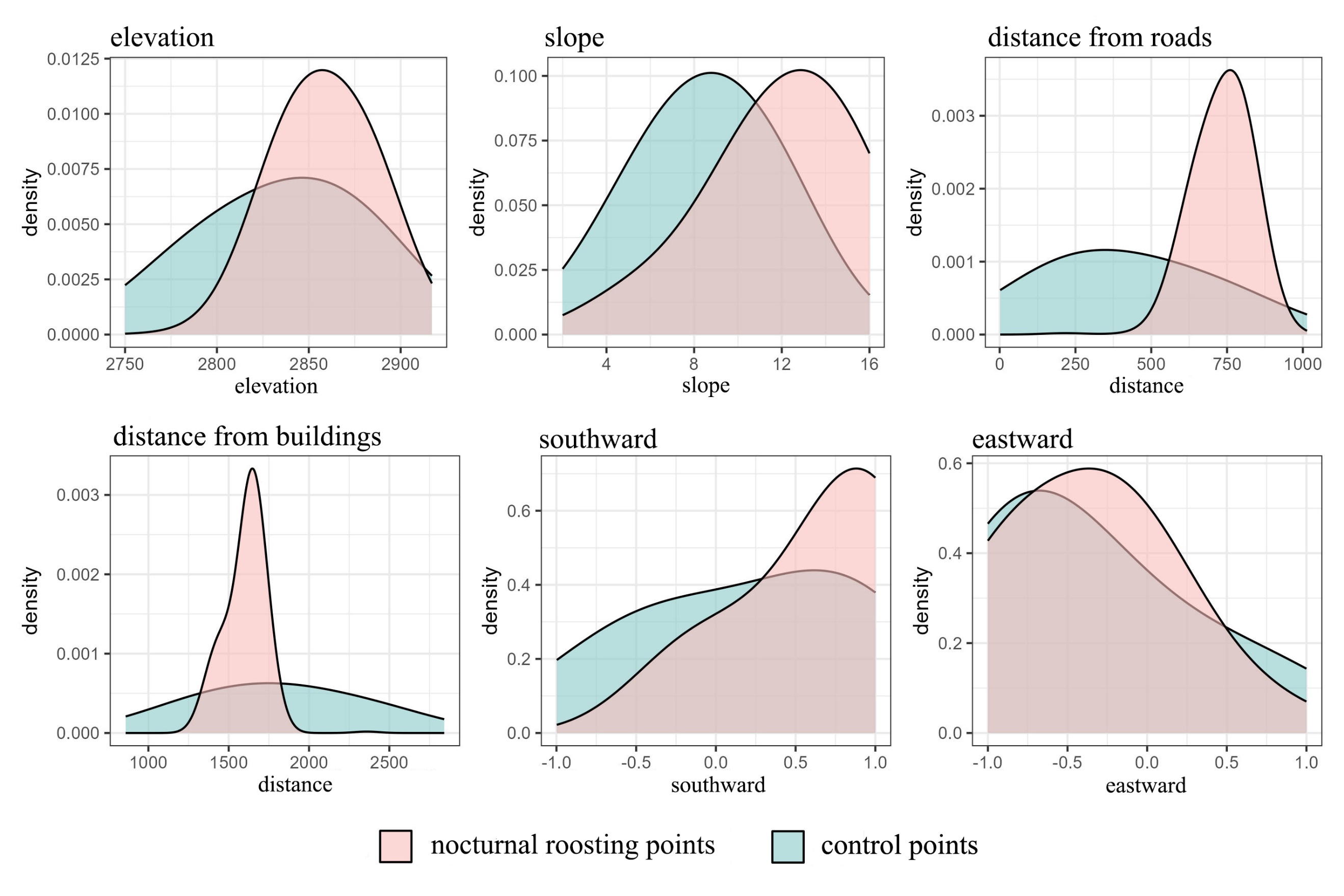


a

c

b

c

a

b

Figure S2 High-resolution image maps

(a: Yongshan, b: Ludian, c: Xundian)


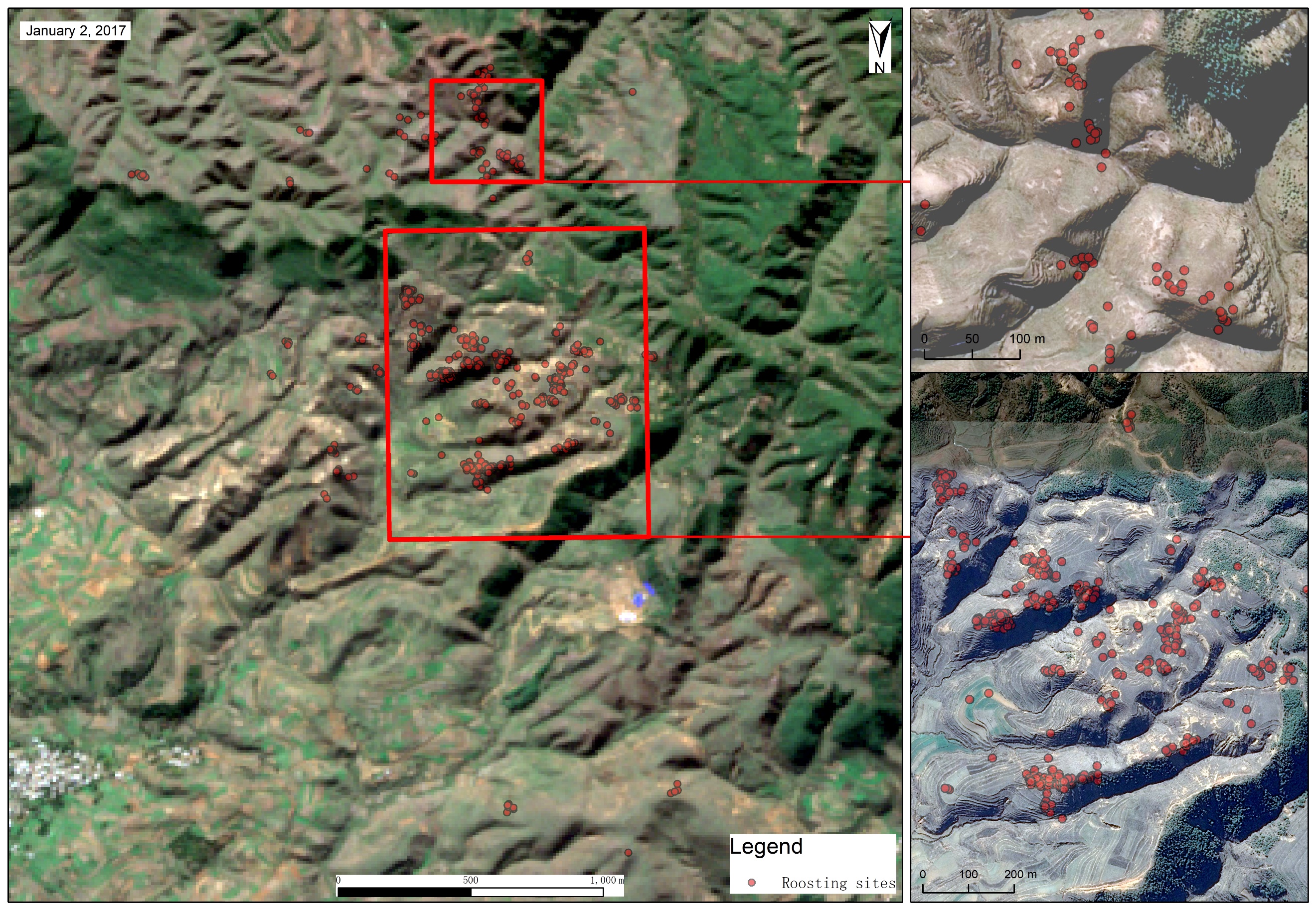

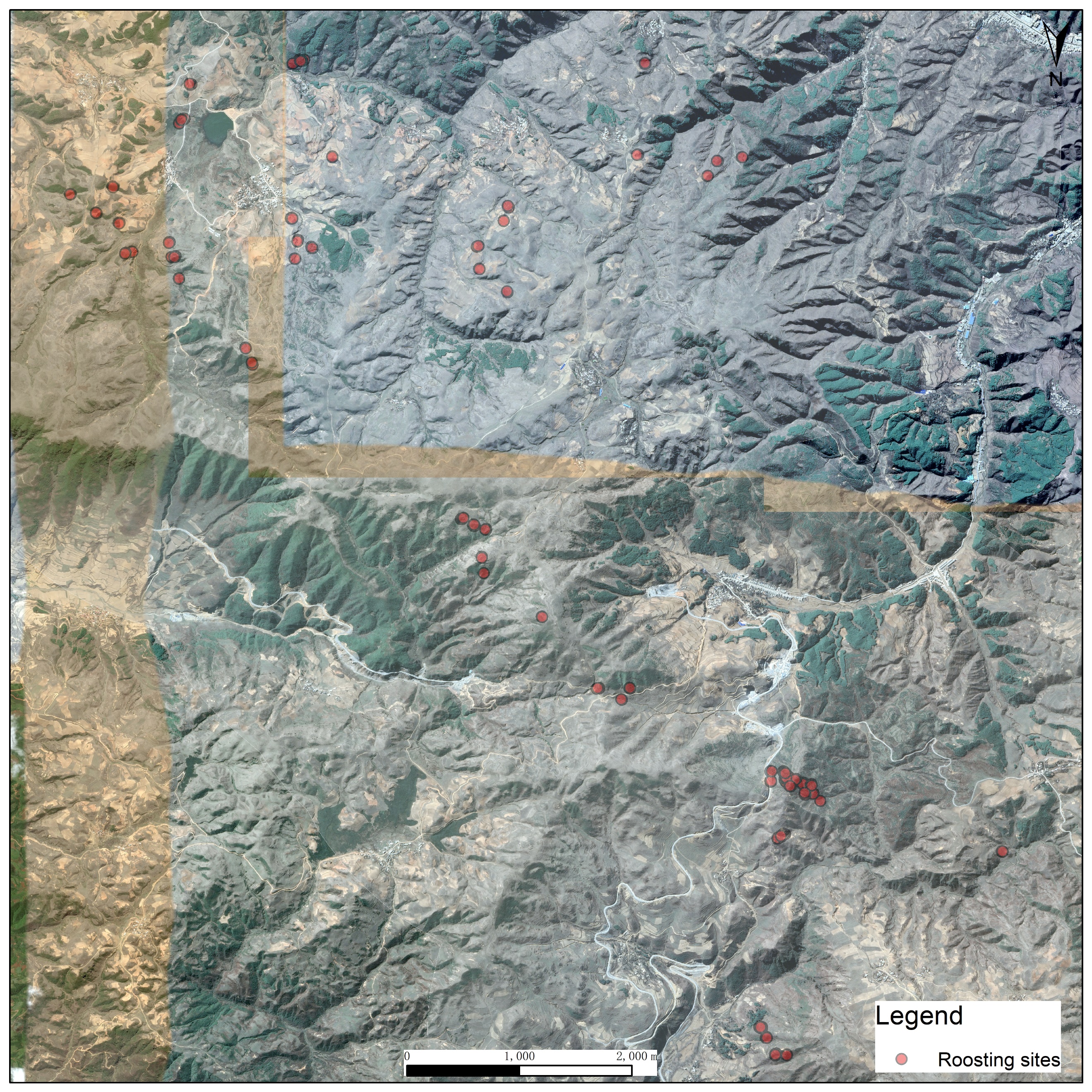

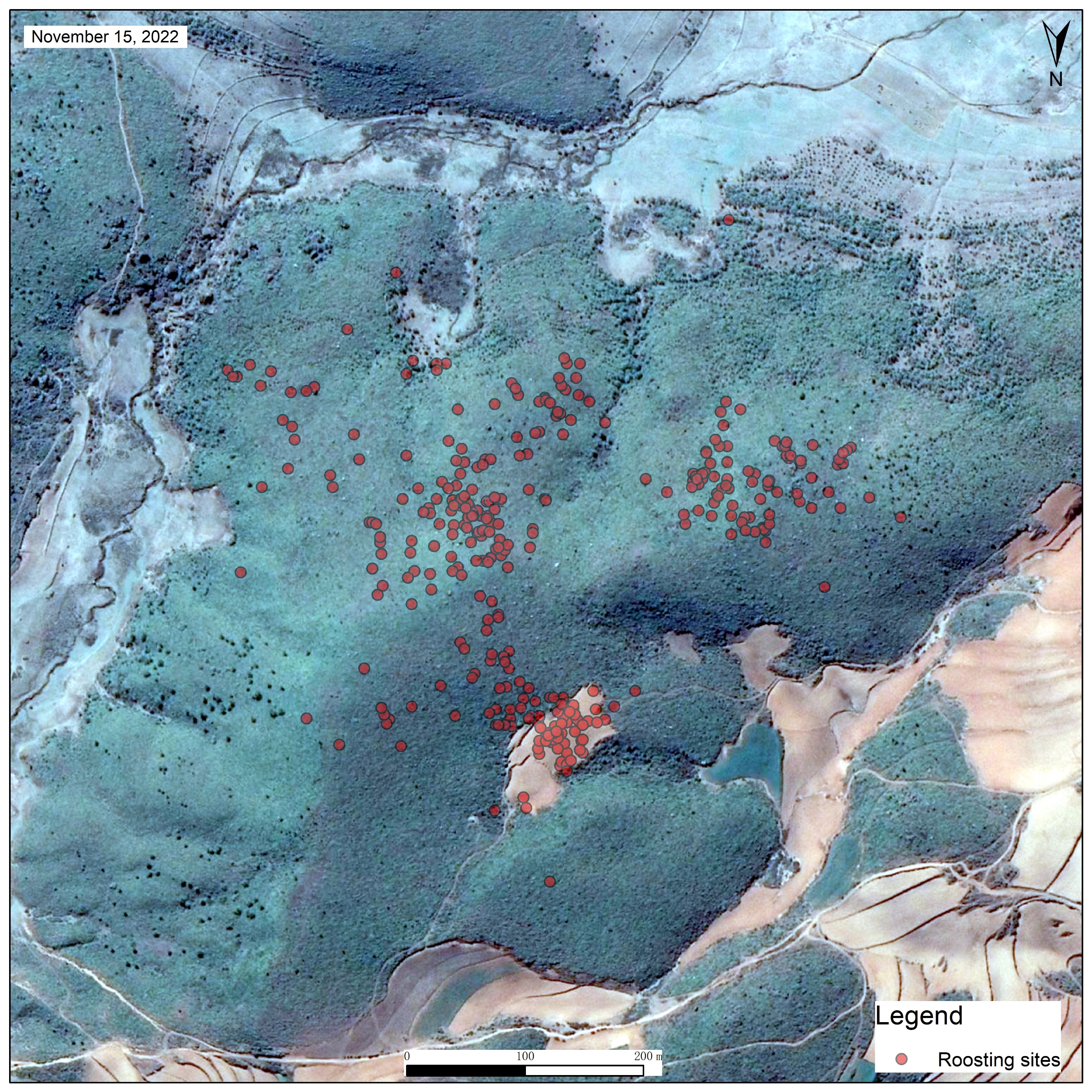


Table S1 Monthly data of black-necked cranes

| Individual | Time |
| --- | --- |
| BNC01 | 2016.11-2017.03 |
| BNC02 | 2016.11-2017.03 |
| BNC03 | 2016.11-2016.12, 2017.03-2017.04 |
| BNC04 | 2016.01, 2016.03 |
| BNC05 | 2016.01-2016.03 |
| BNC06 | 2018.01-2018.03 |
| BNC07 | 2019.01-2019.03 |
| BNC08 | 2021.03-2021.04, 2021.11-2022.03 |
| BNC09 | 2021.11-2022.03 |
| BNC10 | 2021.03, 2021.11-2022.03, 2022.11-2023.03, 2023.11-2023.12 |
| BNC11 | 2020.12-2021.03 |
| BNC12 | 2021.03-2021.04, 2021.11-2022.03 |
| BNC13 | 2022.02-2022.04, 2022.11-2023.03, 2023.11-2023.12 |
| BNC14 | 2022.03, 2022.11-2023.04, 2023.11-2023.12 |

**Study design.**

We investigated whether temperature and wind direction influence the selection of terrestrial nocturnal roosting sites by black-necked cranes. To reduce potential errors caused by spatial variability in meteorological conditions, we focused our analysis on Ludian, the area closest to the Zhaotong meteorological station (approximately 30 km away). We collected meteorological data at three-hour intervals from the Zhaotong station, using records published by the National Climatic Data Center (NCDC, ftp://ftp.ncdc.noaa.gov/pub/data/noaa/isd-lite/) for the period from January to March 2017.

**Statistical Analysis.**

We assessed the response pattern of temperature to the elevation of black-necked crane roosting sites using a linear regression model. To examine the directional relationship between slope aspect and wind direction, we applied circular correlation analysis using the ‘*cor.circular()*’ function from the ‘circular’ R package (Agostinelli and Lund, 2024).

**Result.**

The elevation and temperature of black-necked crane terrestrial roosting sites were not significantly related (t = 0.95761, df = 70, p = 0.342; Figure S3a). Similarly, no significant circular correlation was found between slope aspect and wind direction (r circular = 0.034; Figure S3b).

Figure S3 (a) Relationship between temperature and the elevation of black-necked crane terrestrial roosting sites (b) Relationship between wind direction and the slope aspect of black-necked crane terrestrial roosting sites


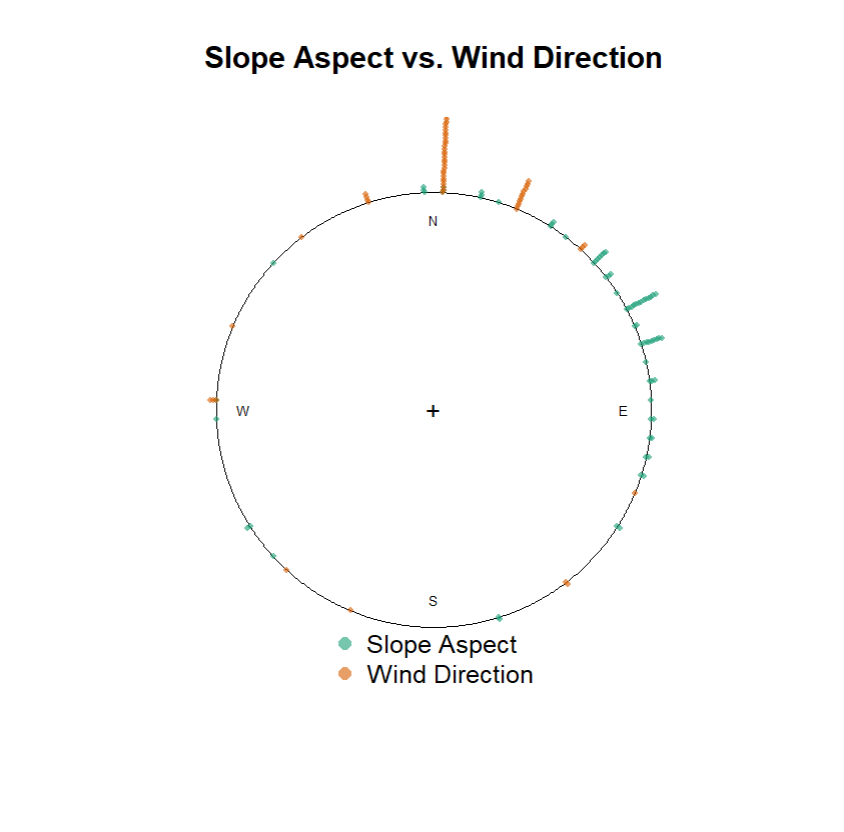


b


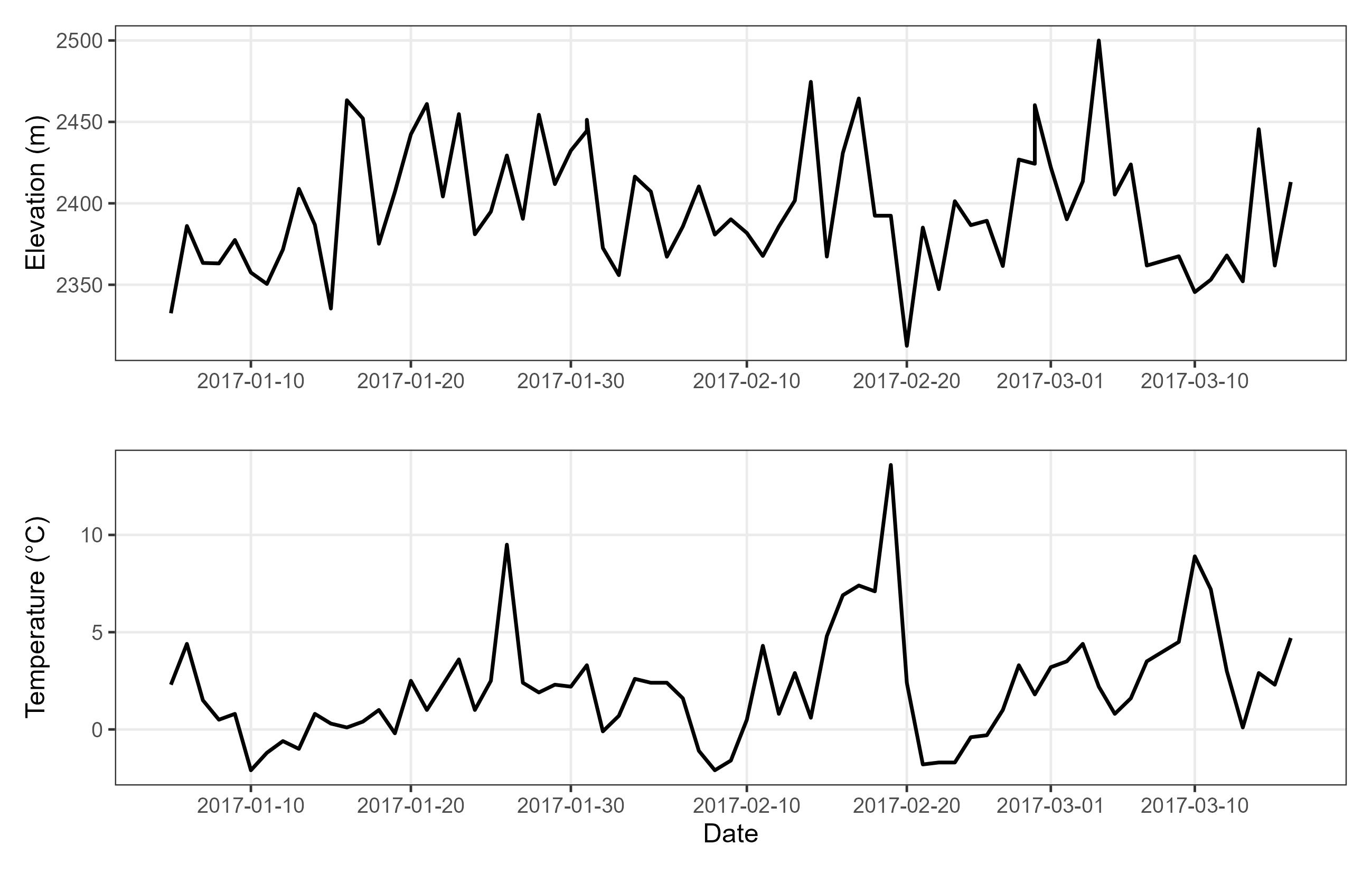


a

**References.**

Agostinelli, C. and Lund, U. (2024). R package 'circular': Circular Statistics (version 0.5-1).
